# Massive lower gastrointestinal bleeding due to rupture of the superior mesenteric artery branch in Behçet’s disease: case report

**DOI:** 10.3389/fimmu.2025.1668761

**Published:** 2025-12-05

**Authors:** Jing-fen Ye, Jian-long Guan

**Affiliations:** 1 Department of Rheumatology and Immunology, Huadong Hospital Affiliated to Fudan University Shanghai, Shanghai, China; 2 Department of Rheumatology and Immunology, Huadong Hospital, Fudan University, Shanghai, China

**Keywords:** vasculitis, Behçet’s disease, mesenteric artery, rupture, computed tomography angiography, endovascular embolization treatment

## Abstract

**Background:**

Behçet’s disease (BD) is an enigmatic autoimmune vasculitis that affects vessels of all sizes, causing corresponding symptoms in multiple organ systems. Arterial lesions in patients with BD are rare and generally involve large arteries, such as the aorta, the pulmonary artery, and the femoral arteries. Arteriorrhagia due to the mesenteric artery is a potentially life-threatening condition. To date, there are very few reports of this condition.

**Case presentation:**

We report on a 41-year-old male patient with a 4-year history of intestinal BD. He presented with atypical abdominal pain followed by massive lower gastrointestinal bleeding. The initial suspicion was a recurrence of intestinal BD. Endoscopy revealed esophageal and anastomotic ulcers. After conservative medical treatment, the disease progressed to persistent bleeding after 8 days of hospitalization. Computed tomography angiography revealed extravasation around a branch artery of the superior mesenteric artery. Following selective embolization of the superior mesenteric artery, the gastrointestinal bleeding stopped completely within a few days. After this procedure, the patient was treated with immunosuppressive therapy without any bleeding to date.

**Conclusion:**

This is a rare case in which recurrent abdominal pain and aggressive gastrointestinal bleeding were due to a rupture of the superior mesenteric artery branch, requiring a differential diagnosis from intestinal BD. A prompt endovascular intervention therapy may be lifesaving. Early initiation of immunosuppressive therapy is indicated after surgical treatment, given the inflammatory nature of vascular BD.

## Background

Behçet’s disease (BD) is a chronic inflammatory disorder characterized by its most common clinical manifestations, including oral ulcers, genital ulcers, and skin lesions. Other systems may also be affected, including the ocular, gastrointestinal, articular, neurological, and cardiovascular systems, presenting alone or in various combinations and simultaneously or at different stages of the disease (1). It presents as an alternating process of recurrence and remission. There is no universally accepted laboratory biomarker for BD, and its diagnosis has been heavily based on nonspecific clinical manifestations and the exclusion of other diseases. Thus, the diagnosis and the treatment may be delayed. Some rare cases of BD involving the visceral arteries pose challenges in their diagnosis.

Cardiovascular involvement in BD is typically recognized as a systemic vasculitis that can potentially affect both arteries and veins of almost any organ (2). Vascular involvement occurs in approximately 6.2% of patients with BD in China. Men are more prone than women, and the peak age at diagnosis is 45 years (interquartile range, 36–54 years) (3). The clinical expression is dependent on the affected vessel and the type of injury. Venous involvement, which is primarily manifested as superficial vein and deep vein thrombosis, predominantly affects the upper and lower extremities in up to 70%–80% of patients with vascular BD (2–4). Thrombosis at atypical sites can involve the inferior and superior vena cava, the hepatic veins with Budd–Chiari syndrome, the portal vein, the cerebral venous sinuses, or the right ventricle (4). Vascular involvement in BD is not rare, while arterial involvement, presenting as thrombosis, stenosis, occlusion, and aneurysm, occurs in only 2%–5% of patients with vascular BD (5). Risk factors include hypertension, smoking, long duration of disease, higher activated partial thromboplastin time (aPTT), and anti-neutrophil cytoplasmic antibody (ANCA) positivity (6–8). Arterial involvement typically succeeds venous involvement, which usually occurs 5–10 years after disease onset.

Nevertheless, it should be remembered that vascular involvement can also occur before fulfillment of the International Criteria for Behçet’s disease (ICBD) criteria. Arterial lesions typically involve large arteries, such as the thoracic aorta, the abdominal aorta, upper limb arteries, lower extremity arteries, the carotid, and the pulmonary artery (2, 4, 5). Uncommon mesenteric artery lesions are challenging to recognize as the index of clinical suspicion is relatively low. Clinical symptoms can range from a casual finding in a completely asymptomatic patient to a very severe and catastrophic picture, such as aneurysm rupture.

Although the rate of mesenteric artery involvement in BD is rare, it is one of the leading causes of death in patients with BD. Massive gastrointestinal bleeding is a rare clinical picture in patients with BD and is usually thought to be from the gastrointestinal mucosal ulcerative lesions, which may lead to misdiagnosis and delay treatment. This case contributes to the limited data available thus far regarding unusual outcomes in vascular BD.

## Case presentation

### Medical history

The patient was a 41-year-old man. He suffered from recurrent oral and genital ulcers at 11 years old. He had a dramatic history of having survived catastrophes caused by aneurysms of the cerebral artery 10 years prior. At the age of 37 years, he underwent right hemicolectomy and appendectomy due to an acute abdomen. Upon operation, macroscopic examination of the resected material showed a large ulcer involving the ileocecal region. Upon pathological examination of the ulcer involving the muscular tissue, considerable lymphocyte infiltration was observed around some arterioles and venules, which revealed signs of vascular inflammation, but not a granulomatous lesion. Intestinal tuberculosis and lymphoma were excluded based on a negative B-cell gene rearrangement and the acid-fast staining results. Thus, a clinical diagnosis of intestinal BD was made, and he was treated with hormones combined with thalidomide and infliximab for 1 year. Unfortunately, the traditional drug regimen was ineffective for him, and he suffered from intestinal obstruction. Therefore, he reluctantly underwent a second enterectomy 1 year after the first resection. He underwent pharmacotherapy, including prednisone, thalidomide, and tofacitinib, after the second surgery. He did not have any oral and genital ulcers, and the postoperative blood results showed no signs of recurrence. However, he still presented with progressively increasing abdominal pain over the last month. Thus, he needed to come to our hospital again for examination and treatment.

### Current hospitalization

At the current admission, physical examination showed tenderness without rebound pain in the right lower quadrant. Laboratory analysis revealed mild inflammatory syndrome, neutrophilia (75.4%), and mild anemia. His C-reactive protein (CRP) was 12 mg/L (typically less than 10 mg/L). The patient’s liver function, kidney function, coagulation function, blood sugar, and blood lipids showed no apparent abnormalities. Infectious workup, including HIV, viral hepatitis, syphilis, G test, and tuberculosis, was negative. Antinuclear antibody, rheumatoid factor, ANCA, and other autoantibodies were all negative. No abnormalities were recognized on chest radiography, electrocardiography, vascular B-ultrasound, and echocardiography. Contrast-enhanced CT of the whole abdomen demonstrated a segmental stenosis with a thickened and enhanced wall of the sigmoid colon. Inflammatory stenosis caused by intestinal BD was considered.

**Figure 1:**
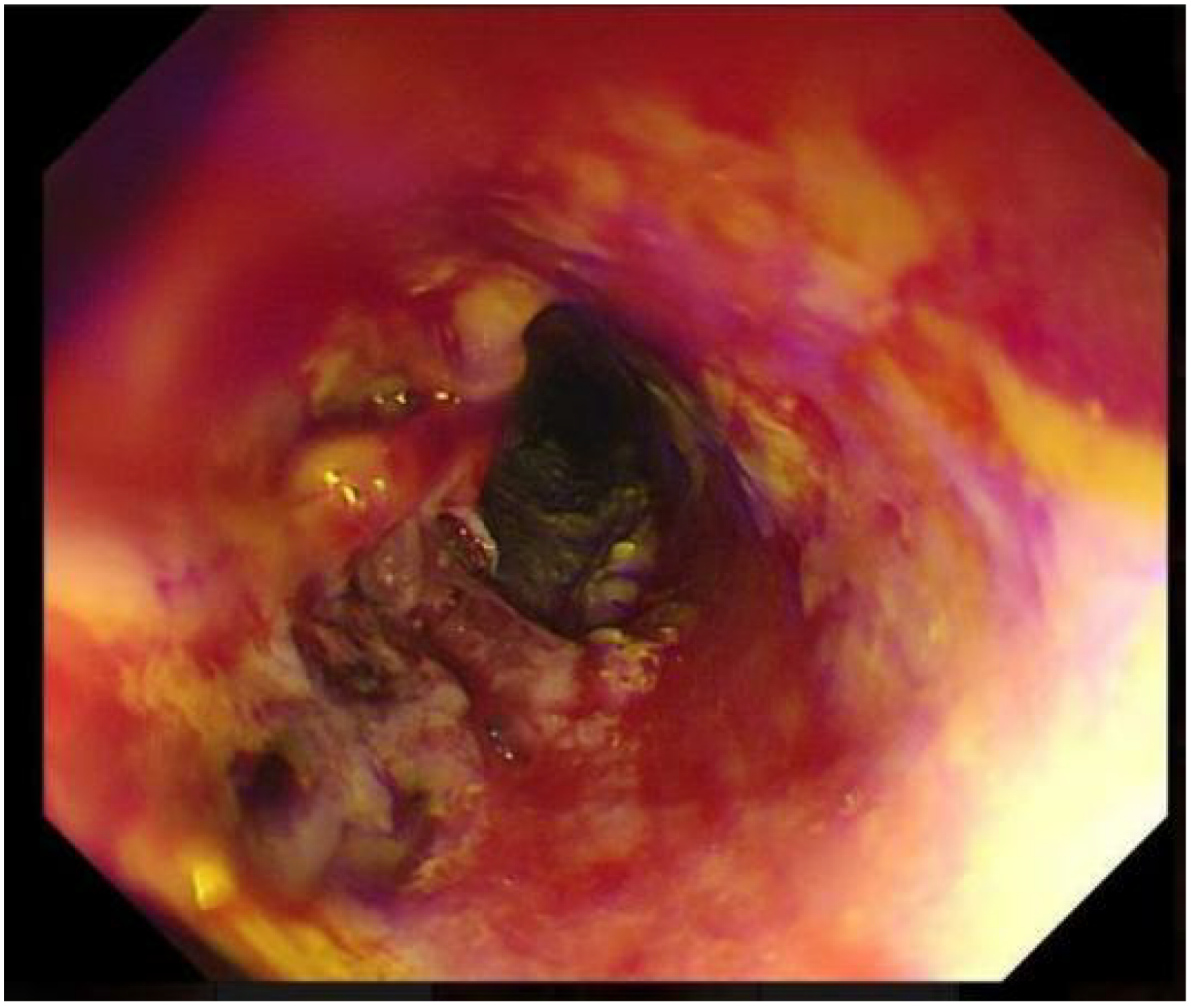
Extensive erosion of the esophageal mucosa, deep ulcer formation, and bleeding under gastroscopy.

**Figure 2:**
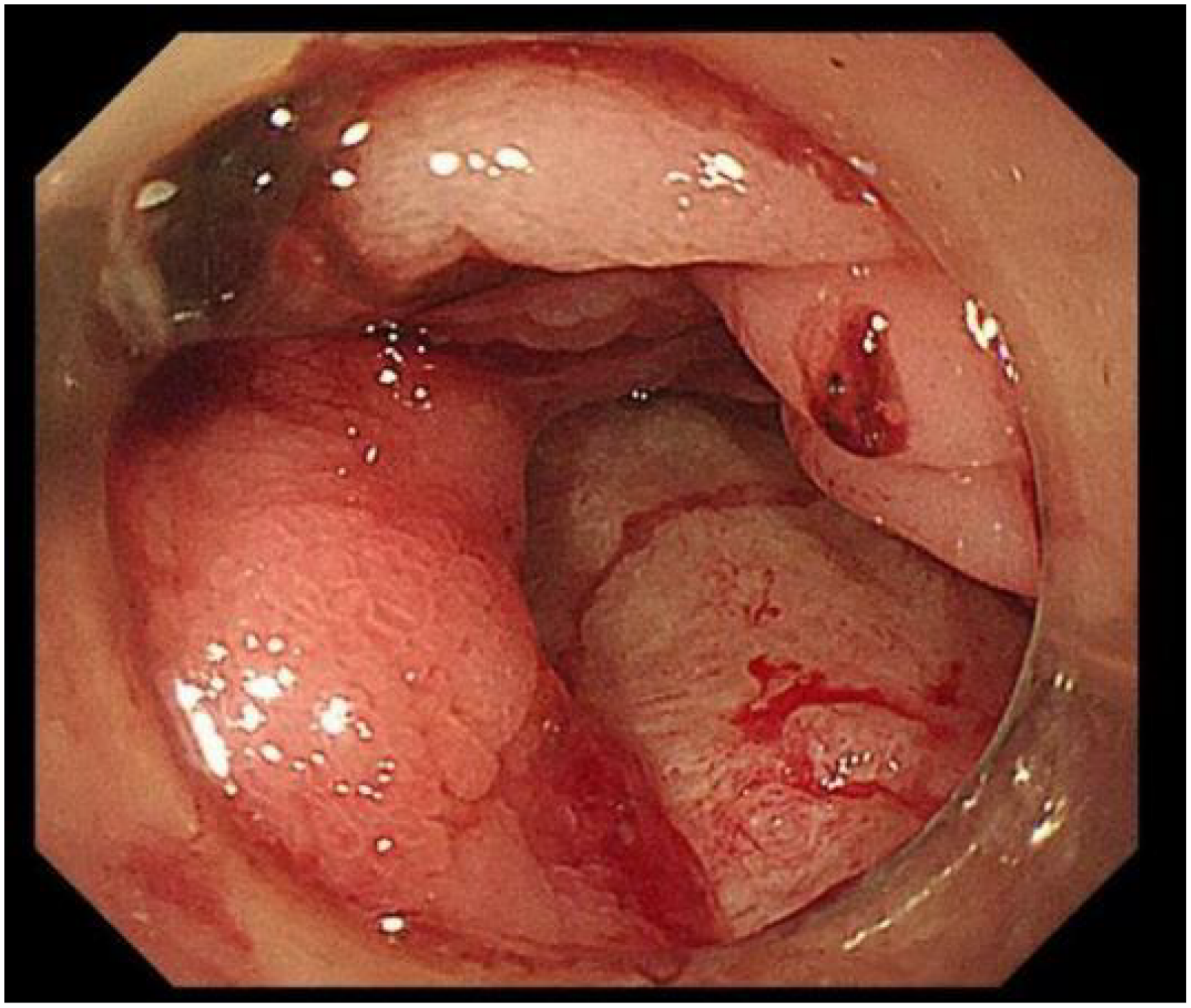
Anastomotic ulcer showing a small amount of bleeding.

**Figure 3:**
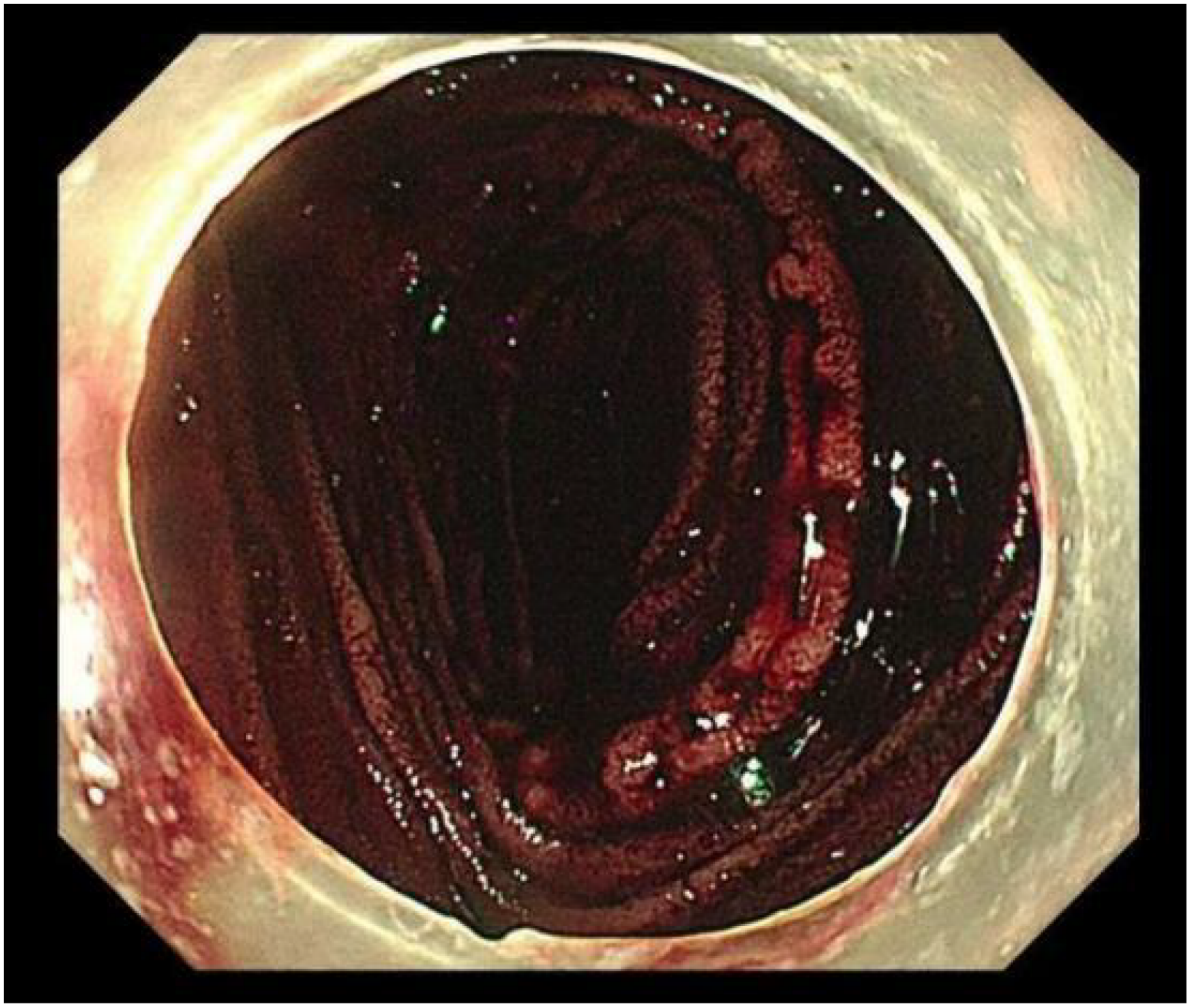
A large amount of fresh blood in the superior anastomosis under colonoscopy.

**Figure 4:**
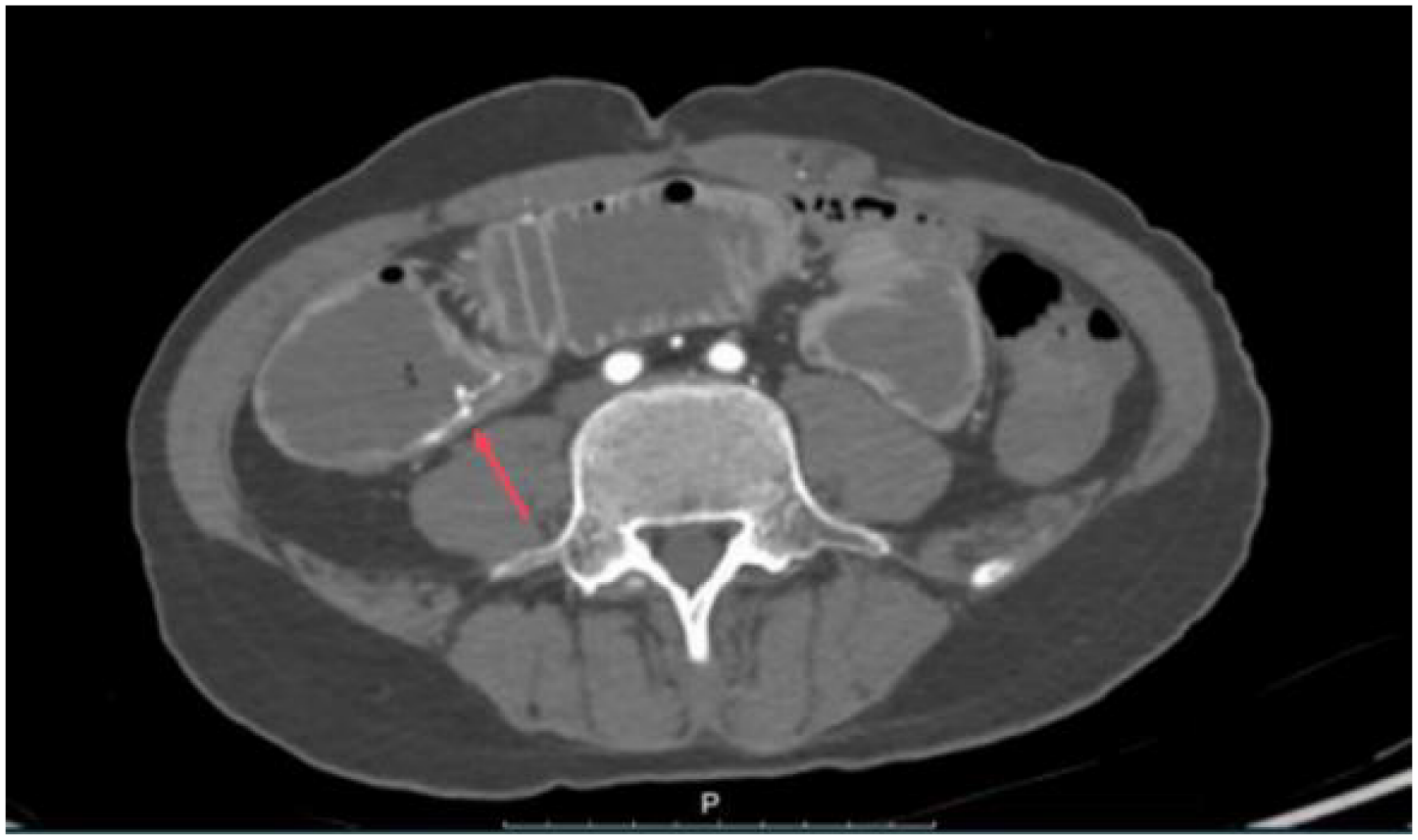
Mesenteric artery computed tomography scan revealing that one branch of the mesenteric artery had apparent extravasation of contrast material.

The patient was started on an oral medication regimen for intestinal BD. However, the medication did not control his abdominal pain. At 8 days after admission, a severe colicky pain in the right lower quadrant of the abdomen started, and he subsequently excreted fresh bloody feces (approximately 600–800 ml of blood). Hemoglobin fell from 107 to 76 g/L, and the patient underwent an emergency gastroscopy and colonoscopy to determine the cause of the bleeding. Gastroscopy revealed extensive erosion of the esophageal mucosa, with deep ulcer formation and bleeding (**Figure 1**). Colonoscopy revealed a large amount of fresh blood in the superior anastomotic segment and a sinus tract formation at the anastomosis, accompanied by a small amount of bleeding (**Figures 2** and **3**). In addition to the anti-inflammatory effects of the immune agent, the anticoagulant drug somatostatin was also administered to improve the bleeding condition. Moreover, blood transfusions and fluid replacement were performed to maintain stable blood pressure. However, the gastrointestinal bleeding did not improve. To investigate the persistent bleeding, the patient was exposed to mesenteric artery CT angiography, which revealed an abnormal signal on the terminal ileum artery with apparent extravasation of contrast material (**Figure 4**). Endovascular therapy was proposed. A guiding catheter was inserted into the mesenteric artery, and superselective embolization of the lesion in the superior mesenteric arterial branch using gelatin sponges was successful. The gastrointestinal bleeding then completely stopped immediately. After this endovascular embolization procedure, he was treated with prednisolone, thalidomide, and adalimumab, without any bleeding. The patient was discharged in good condition after approximately 1 month. Currently, at 6 months of follow-up, there are no signs of relapse.

**Figure 5:**
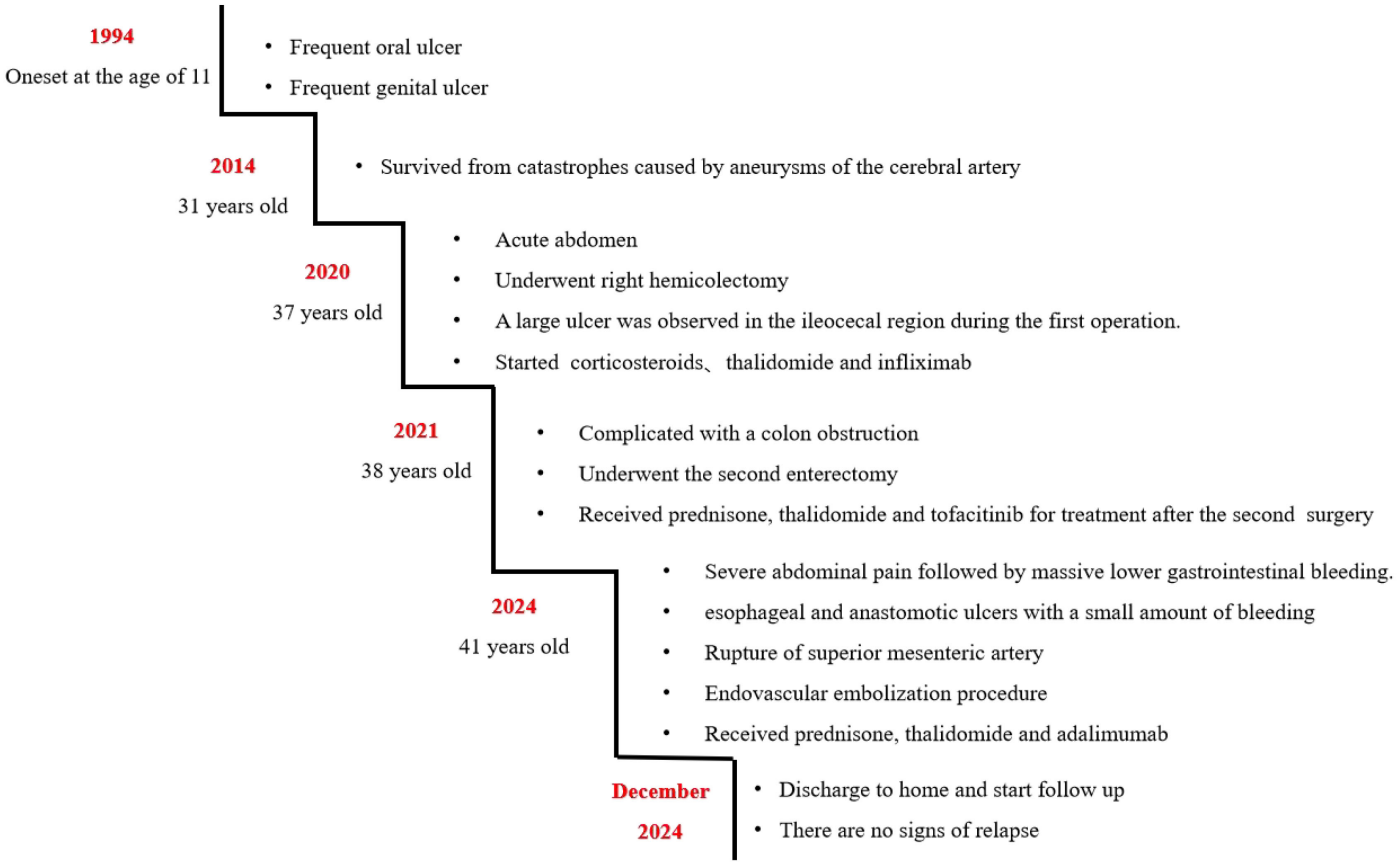
Picture illustrating the disease progression in chronological order.

The timeline of the disease course for this patient is illustrated in **Figure 5**.

### Personal and family history

The patient had no history of smoking or drinking habits, as well as no history of special genetic diseases or congenital vascular diseases.

## Discussion

BD is a multi-systemic syndrome with clinical hallmarks of recurrent oral and genital ulcers and skin lesions (9). It can also affect the gastrointestinal, musculoskeletal, ocular, cardiovascular, neurological, and hematologic systems. Currently, there are no generally accepted diagnostic biomarkers for BD. Thus, the International Study Group Criteria in 1990 and the revised version of the ICBD in 2014 are still the most widely recognized clinical classification criteria. Vascular and neurological manifestations, as the point-rating items, were incorporated into the 2014 ICBD criteria (10). Our patient had repeated oral aphthosis (2 points) and genital ulcerations (2 points) since childhood, a cerebral aneurysm (1 point), and a mesenteric artery lesion (1 point), fulfilling the ICBD criteria.

Gastrointestinal and mesenteric artery involvement can lead to abdominal pain and gastrointestinal bleeding symptoms. However, the colonoscopy examination showed an ulcer. It appears unable to account for the source of the persistent bleeding. Contrast-enhanced CT and enteroscopy ruled out tumors as the source of bleeding. The massive lower gastrointestinal bleeding could be explained by the rupture of the visceral artery. Previous studies have highlighted that atherosclerosis, infection, arteritis, and connective tissue disorders are risk factors for visceral artery rupture (11). The young age, absence of smoking history, diabetes, and dyslipidemia did not suggest an atheromatous origin. Infectious tests and rheumatism indicators were negative. Combined with the BD history, the patient’s only risk factor was vasculitis, and mesenteric arteritis was suggestive of the cause of bleeding. We found two BD patients presenting with lower gastrointestinal bleeding related to mesenteric arterial abnormality in a literature review (12, 13). Similar to previous reports, our case exhibited an overlapping of both the gastrointestinal and vascular phenotypes, which poses a significant diagnostic challenge. This clinical course underscores a diagnostic consideration: intestinal BD that does not show sustained improvement with immunosuppression should prompt deeper investigation for severe bleeding.

There are currently no histopathological features specific to vascular BD. In recent years, imaging techniques have increasingly become an important part of the diagnosis of vasculopathy in patients with BD (14). Doppler ultrasound is an easy-to-access and practical examination. Asymptomatic vascular involvement is a significant concern in the population, especially for BD patients with risk factors of venous vascular events. However, this method is unsuitable for evaluation of the mesenteric artery (12). Contrast-enhanced CT and MR angiography appear to be beneficial in the evaluation of the entire blood vessel system. In the early stages, they can show an irregular wall thickening, fat stranding, and mural contrast enhancement. Stenosis, occlusion, and aneurysm formation are observed in the later stages (15). Their evaluation of different organs has its own advantages and disadvantages.

Both contrast-enhanced CT and MR angiography can be used for the imaging of peripheral arterial involvement in patients with BD. For cerebral venous sinus thrombosis, MR angiography is superior to contrast-enhanced CT for detecting clots in the cortical or deep veins (4). However, thrombosis, dissection, and aneurysm in the abdominal visceral arteries or thrombosis in visceral veins may cause acute abdominal pain. Computed tomography (CT) appears to be the most effective modality for the diagnosis as it provides high detail images in a very short time (16). In our case, the patient was evaluated using contrast-enhanced CT angiography, which revealed a ruptured branch of the superior mesenteric artery. Urgent surgery was performed with superior mesenteric artery embolization, resulting in significant improvement. Such findings are consistent with previous reports on BD-associated aneurysms (17).

Currently, there is no definitive therapeutic modality for patients with vascular BD. Arterial involvement of BD usually requires surgical intervention. There is no consensus regarding the optimal intervention modality. We here described the effectiveness of arterial embolization in a patient with persistent bleeding caused by BD with mesenteric artery involvement. Despite a previous negative clinical trial (18), endovascular treatment is already recommended as a less invasive procedure with rapid and sustained remission. Vascular BD is a condition with an unknown pathogenesis. The most widely accepted hypothesis is that immunological and inflammatory mechanisms cause endothelial damage, leading to subsequent venous thrombosis or arterial wall rupture. Previous studies found that the levels of the biological markers of inflammation, including the neutrophil count, the white blood cell count, the neutrophil-to-lymphocyte ratio (NLR), the erythrocyte sedimentation rate (ESR), D-dimer, and CRP, were significantly increased in patients with cardiovascular involvement (7). Hyperactivated infiltrating neutrophils have been described at the perivascular site and are considered to play a crucial role in the inflammation of vascular BD (19). Neutrophils infiltrating around the vessel wall are activated by infection, resulting in the enhanced production of reactive oxygen species (ROS), which can lead to endothelial damage (4). This pathogenesis directly determines the treatment approach. The clinical significance of high-dose corticosteroids and immunosuppressive therapy is well known. The 2018 European League Against Rheumatism recommendations suggest combining high-dose corticosteroids with cyclophosphamide in the acute phase of BD (20). Moreover, anti-TNFα should be considered for refractory and life-threatening cases of arterial vasculopathy (21). Yamamoto et al. (22) reported on one case experiencing multiple recurrent pseudoaneurysms without immunosuppressive therapy after endovascular repair. In contrast to previous experiences, we administered intensive immunosuppressive therapy before and after endovascular treatment. As expected, the patient responded well, and no postoperative complications have been observed to date. Thus, in addition to endovascular embolization therapy, the management of gastrointestinal bleeding resulting from a rupture of the mesenteric artery in BD also requires corticosteroids and immunosuppressants in order to reduce postoperative recurrence.

## Conclusions

This paper reports on a case of massive lower gastrointestinal bleeding resulting from the rupture of a superior mesenteric artery branch, aiming to provide a novel dimension for considering the cause and the treatment of gastrointestinal bleeding in the future. When there is an unexplained massive lower gastrointestinal bleeding in a patient with BD, vasculo-BD should be considered as a differential diagnosis. Contrast-enhanced CT angiography can be used as the primary diagnostic tool. A combination of surgical and medicinal treatments is required for the treatment of a vasculo-BD patient who was diagnosed with mesenteric artery vasculopathy. We shall continue to monitor the patient closely as BD has a variable clinical course with unpredictable recurrence and remission.

## Data Availability

The original contributions presented in the study are included in the article/supplementary material. Further inquiries can be directed to the corresponding author.
